# Wear Resistance of Medium Carbon Steel with Different Microstructures

**DOI:** 10.3390/ma14082015

**Published:** 2021-04-16

**Authors:** Xue Han, Zhenpu Zhang, Gary C. Barber, Steven J. Thrush, Xin Li

**Affiliations:** 1 Key Laboratory of Automobile Materials of Ministry of Education, School of Materials Science and Engineering, Jilin University, Renmin Street No. 5988, Changchun 130025, China; xhan@oakland.edu; 2 Automotive Tribology Laboratory, Department of Mechanical Engineering, School of Engineering and Computer Science, Oakland University, Rochester, MI 48309, USA; zzhang@oakland.edu (Z.Z.); barber@oakland.edu (G.C.B.); steven.j.thrush.civ@mail.mil (S.J.T.); 3 U.S. Army Combat Capabilities Development Command Ground Vehicle System Center, 6501 East 11 Mile Rd., Warren, MI 48397, USA

**Keywords:** plastic deformation, adhered debris, wear, friction, heat-treatment

## Abstract

In this research, the tribological properties of different microstructures of medium carbon steel produced by either an austempered process or quenched-tempered process are investigated. The as-received samples with annealed microstructure (spherodized) are austempered to obtain a bainite microstructure or quenched-tempered to obtain a tempered martensite microstructure. The tribological performance of these microstructures was studied using a ball-on-disk UMT3 tribometer. The results indicated that both bainite microstructures and tempered-martensite microstructures produced better wear resistance than pearlite microstructures. At the same hardness level, the austempered disk specimens have less cracking due to higher fracture toughness compared to quenched and tempered steel. For the disks, tempered martensite microstructures produced more plastic deformation compared with bainite microstructures. Mild abrasive wear was observed on the harder disks, however, smearing wear was observed on the softer disks. Adhered debris particles were observed on the balls.

## 1. Introduction

Ferrite/pearlite, bainite, martensite, and austenite are common structures in carbon steel and each has different mechanical properties [1]. To improve the service life and durability of medium carbon steel, an understanding of the wear and friction properties of the different microstructures of medium carbon steel during tribological tests is of considerable importance for a number of engineering applications. Although medium carbon steel has a relatively low tensile strength, it is inexpensive and easy to form. Medium carbon steels have carbon content from 0.30% to 0.60%, making them malleable and ductile. Steels with a carbon content of 0.40% to 0.60% are used for rails, railway wheels, and rail axles. Substitutional alloy elements, such as chromium (Cr), manganese (Mn), molybdenum (Mo), and nickel (Ni) are added to these steels to provide a higher hardenability and improve the steels’ ability to be heat-treated including depth of hardening and resistance to softening during tempering [2].

Heat treatment is a simple and low-cost way to produce various microstructures [3]. Austempering is a high-performance isothermal heat treatment that can impart superior performance to ferrous metals. Keough et al. [4] studied the wear performance of carbidic austempered ductile iron (CADI). In their wear tests, they found that CADI samples had better wear resistance than samples made of austempered ductile iron. The mechanical properties of materials can be significantly improved by isothermal heat treatment since it produces the bainite microstructure [5,6,7]. Lower bainite results in higher ductility than upper bainite due to differences in their microstructures [8,9]. The mechanical properties of these steels are also improved via a quenching and tempering process, producing a tempered martensitic microstructure. Generally, steel properties are improved by a quenching and tempering process, because of the precipitation of a fine dispersion of alloy carbides during tempering [10,11].

Recently, many researchers have investigated the tribological properties of medium carbon steel under different test conditions, surface treatments, and lubrication. Shashidhara et al. [12] studied the tribological behavior of AISI1040 using the vegetable oil Jatropha (Jatropha curcas) and mineral oil using a pin-on-disk tribometer. Compared to mineral oil, the Jatropha raw oil reduced both friction and wear for AISI1040 samples. Litoria et al. [13] studied the wear behavior of AISI4140 steel samples with various surface conditions sliding against DLC-coated boronized discs of AISI4140 steel. Singh et al. [14] studied the tribological behavior of shot-peened quenched-tempered SAE-6150 steel specimens. Their results indicated that if an optimized shot peening intensity was utilized, the shot peening significantly reduced the wear rate of the steel.

A few researchers have studied the effect of different microstructures on the tribological performance of medium carbon steel. Han et al. [15] studied the effect of shot peening on the tribological performance of AISI5160 steel austempered under different conditions. The results showed that lower bainite results in higher wear resistance than upper bainite. Chattopadhyay [16] studied the tribological performance of medium carbon steels with pearlitic morphologies versus medium carbon steels with bainitic morphologies. Isothermal annealing was used to produce the pearlitic and bainitic morphologies. The samples were tested with varying normal loads from 20 N to 50 N under dry sliding. The results showed the bainitic microstructures resulted in a lower wear rate compared with the pearlitic microstructures. This was attributed to the higher hardness, higher dislocation density, and much finer distribution of the austenite–cementite aggregate phase throughout the bainite. In the present study, three different temperatures of isothermal heat treatment were employed to produce bainite microstructures and quenching, and three different temperatures of tempering were employed to produce tempered martensite microstructures using AISI6150 steel specimens. The temperatures of both treatments were chosen in a range to achieve a comparable hardness of the microstructures for both austempered and quenched-tempered samples. A UMT3 wear test machine was employed to study the tribological behavior of bainite and tempered martensite microstructures using a ball on disk sliding configuration. The wear volume and roughness were investigated using 3D profiles. A scanning electron microscope (SEM) was used to observe the surface after tribo-tests.

## 2. Experimental Procedure

### 2.1. Material

**Figure 1:**
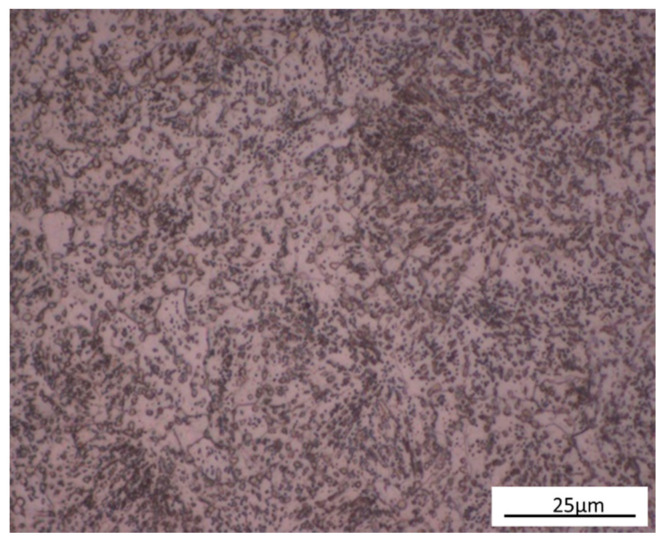
Microstructure of As-Received AISI6150 steel.

**Figure 2:**
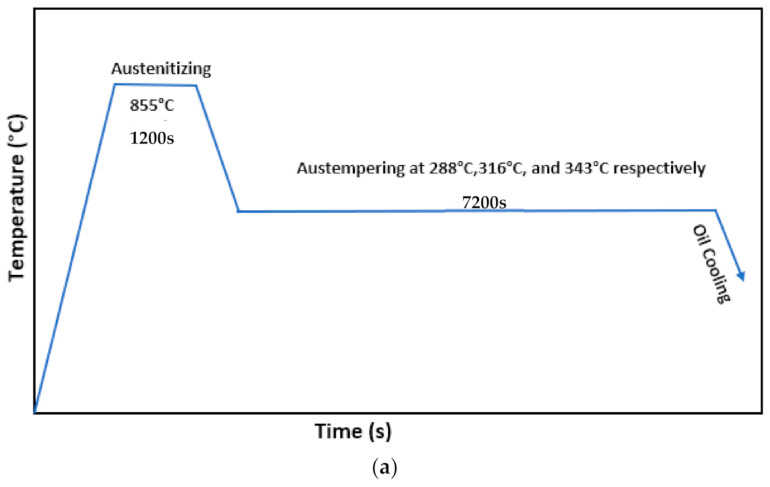

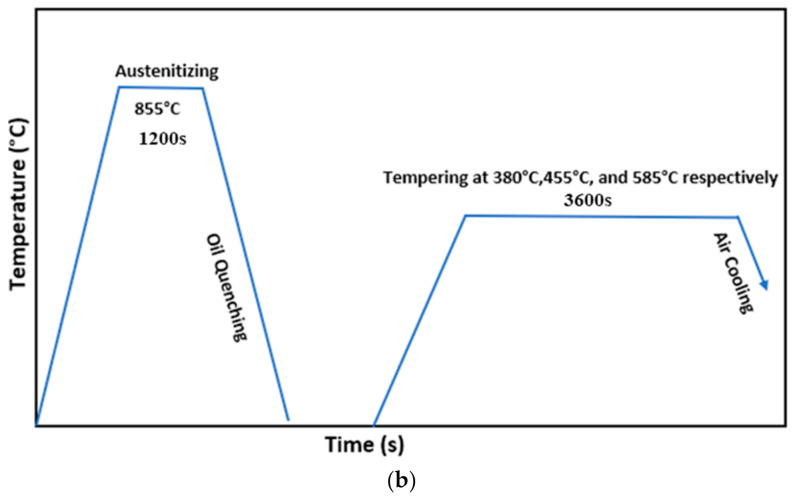
Schematic description of (**a**) austempering process, and (**b**) quenching-tempering process.

The chemical composition of the AISI6150 steel is given in Table 1. As-received samples were mounted and etched using 3% nital and then an optical microscope was used to observe the microstructures. The as-received disks were found to consist of annealed microstructure (spherodized) containing fine spherical carbides and ferrite, see Figure 1. The samples were cut all cut from the same bar of steel and machined into a round shape with a diameter of 30 mm and a thickness of 3.2 mm prior to heat treatment. The samples were austenitized at 855 °C for 20 min by immersing in a salt bath and then transferred quickly to another lower temperature salt bath maintained at 288 °C, 316 °C, or 343 °C respectively, for austempering. Then, the samples were cooled in oil to room temperature. After austempering, 288 °C austempered disks had a hardness of 47.7 HRC, 316 °C austempered disks had a hardness of 45.6 HRC, and 343 °C austempered disks has a hardness of 38.7 HRC, see Table 2. The quenched and tempered disks were also austenitized at 855 °C for 20 min by immersing in the salt bath and then quenched in oil. After quenching, to obtain the same hardness as the 288 °C, 316 °C, and 343 °C austempered disks, the disks were tempered for one hour at 380 °C, 455 °C, and 585 °C. A salt bath is an ideal heat treatment medium because when disks are immersed in a salt bath, air cannot contact the workpiece, therefore, oxidation and decarburization are prevented. A schematic diagram of the austempered and quenched and tempered heat treatments is shown in Figure 2. After the heat-treatment process, the volume fraction of retained austenite was found using X-ray diffraction (XRD). A Bruker D8 XRD (Madison, WI, USA) instrument using Cu radiation at 40 kV and 40 mA current were employed to perform the measurements. The step scan was 0.04 and the speed was 2 K/min.

### 2.2. Tribology Behavior

Tribology testing of the austempered and quenched-tempered AISI6150 disks was carried out with a UMT3 reciprocating ball-on-disk wear tester at room temperature. This tribo-tester slides a ball specimen against a disk specimen with a constant normal load and fixed reciprocation rate. The ball specimens, with a diameter of 9.5 mm, were made of AISI 52100 steel with 60 HRC and 0.2 µm arithmetic average roughness, Ra. To achieve the hardness of 60HRC, AISI52100 specimens were austenitized at 840 °C +/− 5 °C for 15 min, immersed in a salt bath, and then oil quenched to room temperature. The specimens were then tempered at 180 °C for one hour. The disks with a surface roughness of 80 nm ≤ Ra ≤ 150 nm were immersed in PAO4 (Synfluid, TX, USA) oil during testing. A normal load of 320 N was applied to load the ball against the disk with a cyclic speed of 2 Hz and a displacement of 4 mm. The test duration was 10 min. Both disks and balls were degreased and cleaned thoroughly in acetone prior to, and after tests. For each experiment, a new ball and a new disk were used. At least three tests were carried out for each type of disk.

The coefficient of friction was automatically monitored and collected by the UMT3 (Bruker, San Jose, CA, USA) wear tester. All the coefficient of friction data was averaged for each test, and three tests were conducted for each disk at the same test condition. Three different locations of the disk wear scars were measured by the 3-D profiler and averaged for one disk to obtain the average cross-sectional area of the wear scar. Then, the average wear area was multiplied by the wear scar length of 4 mm to obtain the wear volume. The wear scars and subsurface damaged zone were observed by optical microscopy, 3D profiles, and scanning electron microscopy (SEM).

## 3. Results and Discussion

### 3.1. Microstructure and Characterization

**Figure 3:**
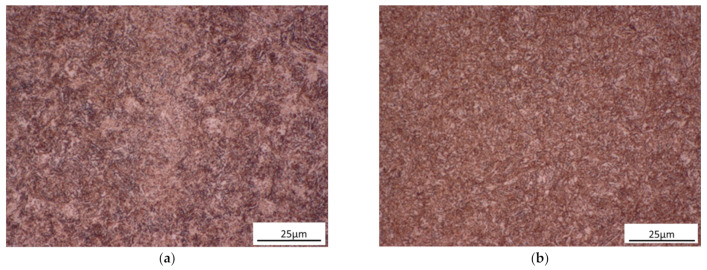
Optical microscopy of the microstructure of AISI6150 steel: (**a**) 343 °C austempered disks, (**b**) 585 °C quenched-tempered disks.

**Figure 4:**
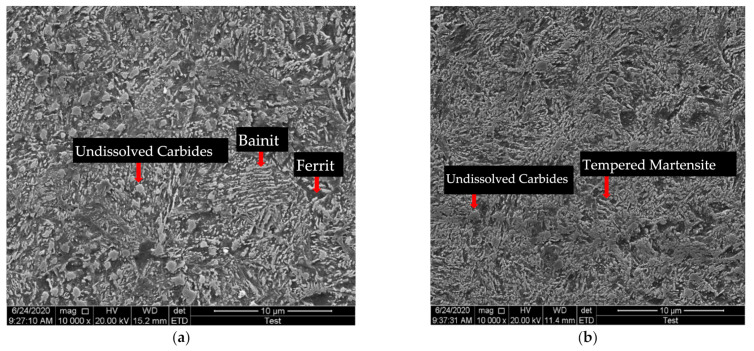
SEM images of microstructure of AISI6150 steel: (**a**) 343 °C austempered disks, (**b**) 585 °C quenched-tempered disks.

Optical microscopy was employed to observe the microstructures. Needle-like bainite was formed when austempering at 288 °C, 316 °C, and 343 °C. Tempered martensite was formed when tempering at 380 °C, 455 °C, and 585 °C. The microstructures of 343 °C austempered disks and 585 °C quenched-tempered disks are shown in Figure 3. Undissolved carbides were observed in the 343 °C austempered disks and 585 °C quenched-tempered disks, see Figure 4. The SEM image of 343 °C austempered disks shows that ferrite precipitates at the grain boundaries and needle-like bainite was formed, see Figure 4a. A high fraction of carbides and tempered martensite were observed on the 585 °C quenched-tempered disks, see Figure 4b.

### 3.2. The Volume Fraction of Retained Austenite

Before the tribo-tests, the volume fraction of retained austenite was determined by using XRD. Table 3 indicates that all the heat-treated disk samples contained only small fractions of retained austenite.

### 3.3. Hardness

The Rockwell hardness for each disk was measured at three different locations for each disk. Since there were three disk specimens for each type of heat treatment, a total of nine hardness values were measured and averaged. Depending on the Rockwell scale utilized, the range of hardness values for each type of sample was +/−2 HRC or +/−2 HRB. After the austempering process, all the disks had higher hardness than the as-received disks. As the austempering temperature increases, the disks had lower hardness as shown in Table 2. As the tempering temperature was increased for the quench-tempered disks the hardness also decreased. Tempering temperatures were selected based on previous experience and trial and error to produce hardness values similar to the hardness values obtained for the three austempering tempering temperatures as shown in Table 2.

### 3.4. Friction Performance

**Figure 5:**
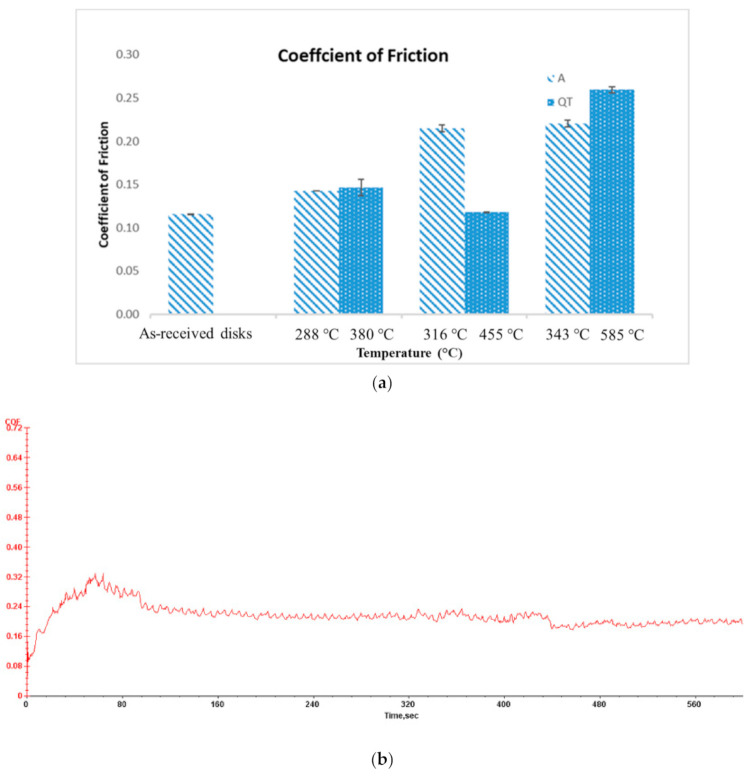
(**a**) Coefficient of friction of austempered disks and quenched-tempered disks; (**b**) COF vs. sliding time for 288 °C austempered disk.

Figure 5 shows the average coefficient of friction and standard deviation of austempered disks versus quenched-tempered disks. To find the average COF (coefficient of friction), first, the mean COF was found for a given test. Then, the average COF for a given heat treatment was found by taking the average of the mean COF for the 3 repeat tests. The standard deviation was calculated using the average COF and mean COF for each of the three repeat tests conducted for specimens with the same heat treatment. All the heat-treated disks produced a higher coefficient of friction than that of as-received disks, except for the 455 °C quenched-tempered disks which produced a similar coefficient of friction as the as-received disks. For most cases, as the austempered or quenched-tempered temperature increased, the coefficient of friction also increased. At the same hardness level, the quenched-tempered disks produced a higher coefficient of friction than austempered disks except for 455 °C quenched-tempered disks. A typical plot of COF versus sliding time is shown in Figure 5b. For the 288 °C austempered disk, the beginning 80 s is the running-in stage with the maximum COF reaching a value of 0.32. After the peaks on the sliding surfaces were removed during the run-in, the COF reached a steady-state value in the range of 0.20 to 0.23.

### 3.5. Wear Performance

**Figure 6:**
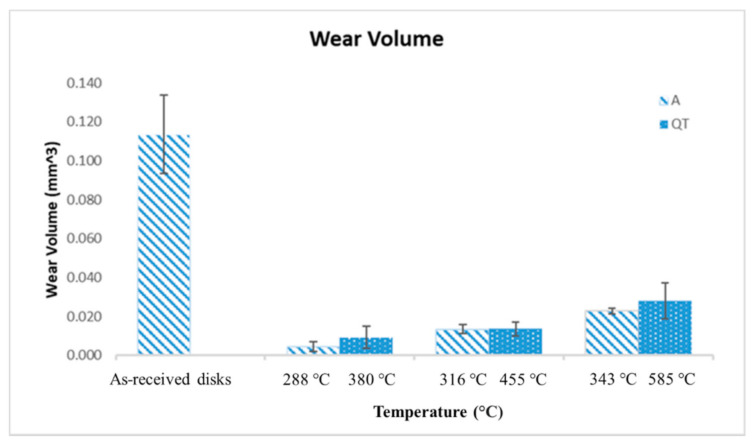
Wear volume of austempered disks and quenched-tempered disks.

Figure 6 shows the average wear volume and standard deviation of austempered disks versus quenched-tempered disks. The average wear value was calculated by averaging the wear value for the three repeat tests for a given heat treatment. The standard deviation was calculated using the average wear value and wear value for each of the three repeat tests conducted for specimens with the same heat treatment. All heat-treated disks produced lower wear volume than the as-received disks. The 288 °C austempered disks and 380 °C quenched-tempered disks had the lowest wear volume as compared to the other austempered and quenched-tempered disks, this is due to their high hardness. As the austempered or quenched-tempered temperature increases, the wear volume increased.The quenched-tempered disks overall had slightly higher wear volume than that of austempered disks at the same hardness level.

### 3.6. Pile Up

**Figure 7:**
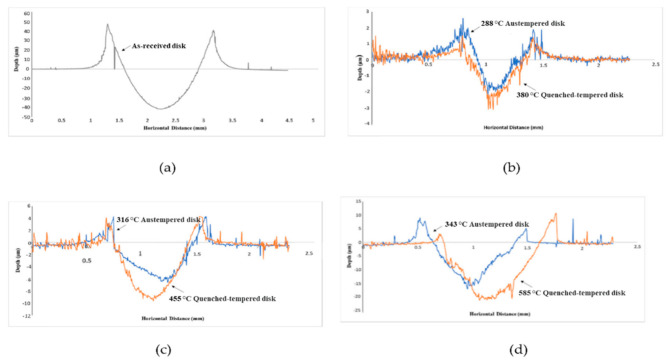
Pile up of: (**a**) as-received disks, (**b**) 288 °C austempered disks versus 380 °C quenched-tempered disks, (**c**) 316 °C austempered disks versus 455 °C quenched-tempered disks, (**d**) 343 °C austempered disks versus 585 °C quenched-tempered disks.

Figure 7 shows the pile up beside wear tracks of as-received disks and austempered disks versus quenched-tempered disks. The pile-up was produced during the tests, due to plastic deformation leading to material flow. 3D profiles were used to measure the amount of pile-up. The total wear volume loss contributed to pile up and the remaining wear volume loss resulted in wear debris. The 288 °C austempered disks generated lower wear loss and higher pile-up compared with the 380 °C quenched-tempered disks. Therefore, 288 °C austempered disks produced less wear debris than 380 °C quenched-tempered disks, see Figure 7b. The 316 °C/343 °C austempered disks generated lower wear loss compared with the 455 °C/585 °C quenched-tempered disks. However, 316 °C/343 °C austempered disks generated a similar pile-up as the 455 °C/585 °C quenched-tempered disks. Hence, 316 °C/343 °C austempered disks also produced less wear debris than 455 °C/585 °C quenched-tempered disks, see Figure 7c,d. Figure 7a shows that the as-received disk generates the highest wear loss and highest pile-up as compared to the heat-treated disks. Therefore, the bainite microstructures and tempered martensite microstructures had higher wear resistance than the annealed microstructure (spherodized) microstructures because of the higher hardness.

### 3.7. Ball Surface

**Figure 8:**
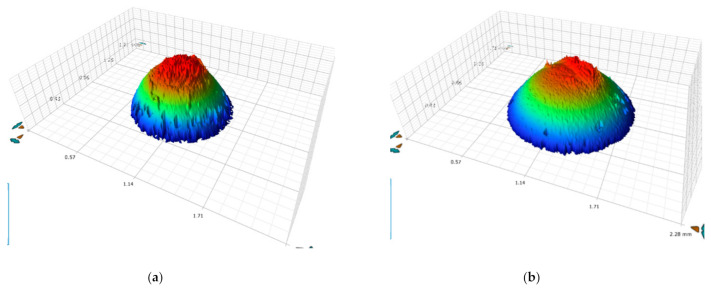

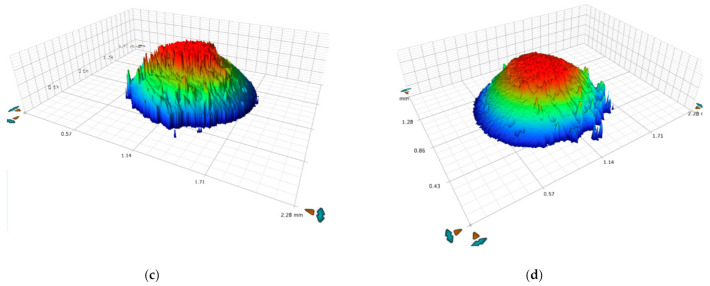
3D profiles showing the wear on the: (**a**) ball run with 288 °C austempered disks, (**b**) ball run with 380 °C quenched-tempered disks, (**c**) ball run with 343 °C austempered disks, and (**d**) ball run with 585 °C quenched-tempered disks.

Figure 8 shows the 3D profiles for balls run with the 288 °C austempered disks, 343 °C austempered disks, 380 °C quenched-tempered disks, and 585 °C quenched-tempered disks. Adhered material was observed on the balls which came from the austempered or quenched-tempered disks. No detectable wear was observed on the balls.

### 3.8. SEM Observations

**Figure 9:**
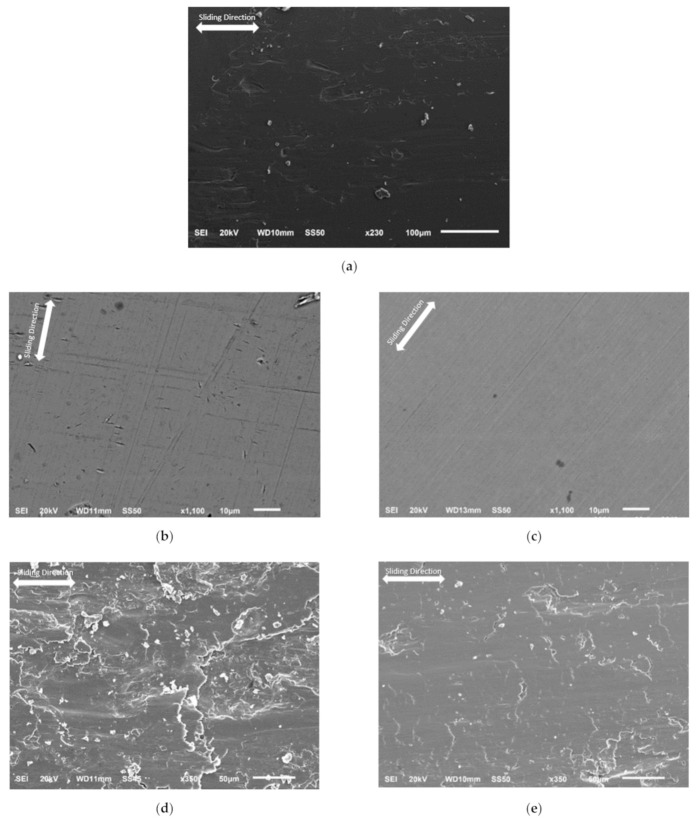
SEM observations on the: (**a**) as-received disks, (**b**) 288 °C austempered disks, (**c**) 380 °C quenched-tempered disks, (**d**) 343 °C austempered disks, and (**e**) 585 °C quenched-tempered disks.

**Figure 10:**
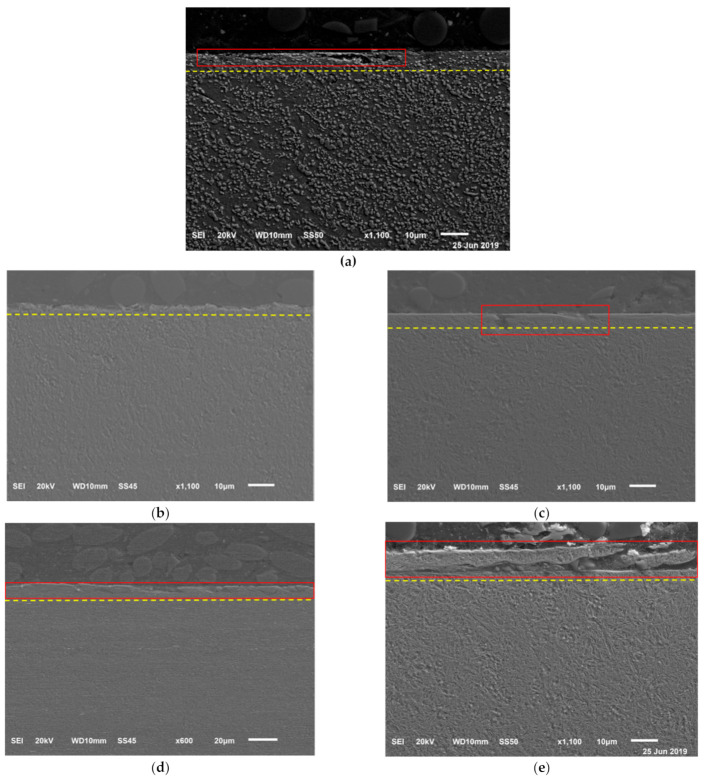
SEM observations on the cross-sections of (**a**) as received disks, (**b**) 288 °C austempered disks, (**c**) 380 °C quenched-tempered disks, (**d**) 316 °C austempered disks, and (**e**) 455 °C quenched-tempered disks. The red lines indicate cracks and the yellow line indicates the plastic deformation zone.

After tribo-tests, the SEM observations of the worn surfaces are shown in Figure 9. Obvious wear debris, plastic deformation, and smearing wear were observed on the as-received disks, 343 °C austempered disks, and 585 °C quenched-tempered disks. The ball with 60 HRC is significantly harder than the as-received, 343 °C austempered, and 585 °C quenched-tempered disks; see Table 2. The material near the surface of the disks is plastically deformed, resulting in smearing wear and resulting wear debris. Some of the debris adhered to the balls. The wear debris led to an increase in wear and the coefficient of friction. The 288 °C austempered disks and 380 °C quenched-tempered disks have high hardness compared with the high temperature austempered or quenched-tempered disks, therefore, only mild abrasive wear was produced during these tests compared to the high temperature austempered or quenched-tempered disks. The consequences of plastic shear deformation due to friction on samples etched with 3% nital are shown in the cross-section views in Figure 10. The plastic shearing produced an alignment of microstructural features in the direction of the sliding close to the surface. The 288 °C austempered disks had the lowest amount of plastic shearing compared with other disks and there are no obvious cracks observed on the cross-section. However, cracks were observed on the as-received disks, 316 °C austempered disks, and 455 °C quenched-tempered disks. This is because the as-received disks, 316 °C austempered disks, and 455 °C quenched-tempered disks have lower hardness compared with the 288 °C austempered disks and 380 °C quenched-tempered disks. Bainite and tempered martensite microstructures have higher wear resistance than the pearlite microstructure. The quenched-tempered disks resulted in more plastic deformation than the austempered disks. At the same hardness level, the austempered disk specimens have less cracking due to higher fracture toughness compared to quenched and tempered steel [17].

### 3.9. Optical Observations

**Figure 11:**
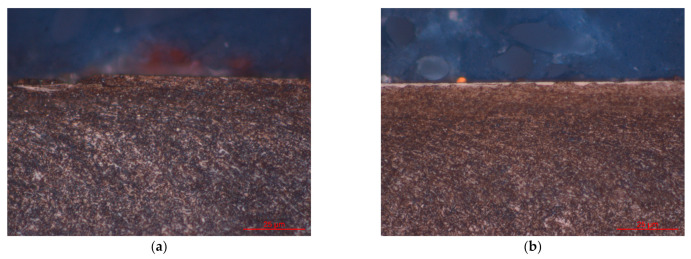
Optical observations on the cross-section of: (**a**) 343 °C austempered disks, (**b**) 585 °C quenched-tempered disks.

A typical white layer was only observed on the 343 °C austempered disk and 585 °C quenched-tempered disk, see Figure 11. The white layer formed because the temperature rises quickly when the disk is in contact with the ball and then quickly drops on the disk when it is not sliding against the ball. Therefore, martensite was formed on the disk surface. However, the martensite is brittle, therefore, cracks are easily formed on the top surface.

## 4. Discussion

The frictional forces generated by the ball specimens sliding against the disks result in high strains generated on the subsurface of the disks [18]. As the tests are run, the plastic deformation zone below the surface will extend, depending on the hardness and microstructures of the samples. Therefore, the softer disks had more significant plastic deformation than the harder disks. At the same hardness level, the austempered disk specimens have less cracking due to higher fracture toughness compared to quenched and tempered steel [17]. Therefore, the austempered disks containing bainite microstructures produced lower wear loss than the quenched-tempered disks containing tempered martensite.

Once the disk microstructures were subjected to very severe plastic strain, the subsurface crack nucleation and propagation lead to wear particles flaking off from the surface. The cracks initiate under the extremely high residual strain gradients which occur in the disk samples [18]. The harder disks have better anti-crack ability than the softer disks. Hence, much more debris and obvious cracks were formed and observed on the softer disks. Harder disks generated fewer cracks, therefore, less wear debris was produced. The particles which flake off from the harder disks act as loose abrasive particles in the tribological system and resulted in mild abrasive wear on the harder disks. The 288 °C austempered disks produced less wear debris than 380 °C quenched-tempered disks, therefore, it generated less wear loss compared with 380 °C quenched-tempered disks. Because the tested balls have much higher hardness than the softer disks the debris adhered to the balls or smeared on the softer disks during the tribo-tests. The debris from the softer disks generated third-body abrasive wear as well. Therefore, 343 °C austempered disks and 585 °C quenched-tempered disks resulted in higher wear loss than other austempered disks or quenched-tempered disks. The tested balls have higher hardness than the tested disks, therefore, there was no obvious wear observed on the ball specimens.

## 5. Conclusions

In the present investigation, the lubricated sliding wear of different microstructures of AISI6150 was studied. The friction and wear regimes were identified. The following points can be highlighted.

1. The tempered martensite microstructure resulted in more plastic deformation and more cracks, due to lower fracture toughness, as compared to the bainite microstructure. This resulted in more debris being produced on the surfaces of samples with tempered martensite microstructure than with the bainite microstructure. Therefore, the tempered martensite microstructure results in higher wear loss than the bainite microstructure.
2. Severe smearing wear was observed on the top surfaces of 343 °C austempered disks and 585 °C quenched-tempered disks. This is because these two disks contain a more ferritic microstructure with low dislocation density which is soft and rather deformable as compared to the other disk specimens which were tested. Therefore, these two disks produced a higher coefficient of friction than the other disks.
3. Less debris was generated on the harder disks resulting in only mild abrasive wear. However, more debris was smeared on the softer disks and third body wear particles were produced resulting in relatively high wear of the softer disks.

## Figures and Tables

**Table 1:** Chemical composition of AISI6150 steel (wt. %).

C	Cr	V	Mn	P	Si	S	Fe
0.51	0.95	0.15	0.80	0.035	0.22	0.04	Balance

**Table 2:** Hardness, HRC, of austempered and quenched-tempered disks after heat-treatment.

Austempered Temperature (°C)	Hardness	Quenched-Tempered Temperature (°C)	Hardness
As received	85.2 HRB	As received	85.2 HRB
288	47.7 HRC	380	48.2 HRC
316	45.6 HRC	455	43.8 HRC
343	38.7 HRC	585	38.3 HRC

**Table 3:** The volume fraction of retained austenite.

Austempered Temperature (°C)	Austempered Retained Austenite (%)	Quenched-Tempered Temperature (°C)	Quenched-Tempered Retained Austenite (%)
288	3.10	380	0.87
316	2.60	455	0.64
343	0.61	585	0.27

## Data Availability

Data sharing is not applicable.

## References

[1] Callister W.D., Rethwisch D.G. (2020). Materials Science and Engineering: An Introduction.

[2] Clarke A.J., Miller M., Field R., Coughlin D., Gibbs P., Clarke K.D., Alexander D., Powers K., Papin A.P., Krauss G. (2014). Atomic and nanoscale chemical and structural changes in quenched and tempered 4340 steel. Acta Materialia.

[3] Zhang J.-C., Zhang T., Yang Y.-T. (2020). Microstructure and properties evolution of Nb-bearing medium Cr wear-resistant cast steel during heat treatment. J. Iron Steel Res. Int..

[4] Keough J.R., Hayrynen K.L. (2000). Carbidic Austempered Ductile Iron (CADI). Ductile Iron News.

[5] Bayati H., Elliott R. (2000). Effect of Microstructural Features on the Austempering Heat Treatment Processing Window. Materials Science Forum.

[6] Shen F.S., Krauss G. (1982). The effect of phosphorous content and proeutectoid carbide distribution on the fracture behavior of 52100 steel. J. Heat Treat..

[7] Jia S.-J., Li B., Liu Q.-Y., Ren Y., Zhang S., Gao H. (2020). Effects of continuous cooling rate on morphology of granular bainite in pipeline steels. J. Iron Steel Res. Int..

[8] Aglan H., Liu Z., Hassan M., Fateh M. (2004). Mechanical and fracture behavior of bainitic rail steel. J. Mater. Process. Technol..

[9] Han X., Zhang Z., Rong Y., Thrush S.J., Barber G.C., Yang H., Qiu F. (2020). Bainite kinetic transformation of austempered AISI 6150 steel. J. Mater. Res. Technol..

[10] Lee W.-S., Su T.-T. (1999). Mechanical properties and microstructural features of AISI 4340 high-strength alloy steel under quenched and tempered conditions. J. Mater. Process. Technol..

[11] Huang D., Thomas G. (1971). Structure and mechanical properties of tempered martensite and lower bainite in Fe−Ni−Mn−C steels. Metall. Trans..

[12] Shashidhara Y.M., Jayaram S.R. (2012). Tribological Studies on AISI 1040 with Raw and Modified Versions of Pongam and Jatropha Vegetable Oils as Lubricants. Adv. Tribol..

[13] Litoria A.K., Joshi A.A., Joshi M.D., Dixit G., Singh D., Hosmani S.S. (2018). Wear behaviour of boronized and duplex-treated AISI 4140 steel against DLC-coated boronized AISI 4140 disc. Surf. Eng..

[14] Singh D., Mondal D.P. (2014). Effect of Quenching and Tempering Processes and shot peening intensity on wear behaviour of SAE-6150 steel. IJEMS.

[15] Han X., Zhang Z., Hou J., Barber G.C., Qiu F. (2020). Tribological behavior of shot peened/austempered AISI 5160 steel. Tribol. Int..

[16] Chattopadhyay C., Sangal S., Mondal K., Garg A. (2012). Improved wear resistance of medium carbon microalloyed bainitic steels. Wear.

[17] Kar R.J., Horn R.M., Zackay V.F. (1979). The effect of heat treatment on microstructure and mechanical properties in 52100 steel. Met. Mater. Trans. A.

[18] Saleski W.J., Fisher R.M., Ritchie R.O., Thomas G., Ludema K.C., Ludema K.C. (1983). The Nature and Origin of Sliding Wear Debris from Steels. Materials & Molecular Research Division, Proceedings of the Conference on Wear of Materials, Reston, VA, USA, 11–14 April 1983.

